# Metastatic colorectal cancer cells upregulate SMLR1 that interacts with tumor-associated macrophages in the liver

**DOI:** 10.47248/chp2401010003

**Published:** 2024-07-18

**Authors:** Chi Wut Wong, Lihua Wang, Jorge Prado Balcazar, Yong Chen, Xiling Shen

**Affiliations:** 1. Terasaki Institute for Biomedical Innovation, Los Angeles, CA 90024, USA; 2. Department of Pharmacology & Cancer Biology, Duke University School of Medicine, Durham, NC 27710, USA; 3. Department of Biomedical Engineering, Pratt School of Engineering, Duke University, Durham, NC 27708, USA; 4. Department of General Surgery, Xiangya Hospital, Central South University, Changsha 410013, China; 5. Department of Pathology, Duke University School of Medicine, Durham, NC 27710, USA

**Keywords:** Colorectal cancer, liver metastasis, small leucine-rich protein 1 (SMLR1), tumor-associated macrophages

## Abstract

Colorectal cancer liver metastasis (CRLM) is the most common form of metastatic colorectal cancer (CRC), one of the leading causes of cancer deaths. The CRLM microenvironment tends to be more immunosuppressive, making immunotherapy less effective. By transcriptomics analysis, we discovered that small leucine-rich protein 1 (SMLR1) is upregulated in CRC liver metastases compared to primary tumors. High SMLR1 expression by the cancer is associated with poor prognosis. Proteomics analysis and cell retention assay revealed associations between SMLR1 and mannose receptor C-type 1 (MRC1, CD206) and sialic acid binding Ig-like lectin 1 (SIGLEC1, CD169) expressed on tumor-associated macrophages in the metastatic liver microenvironment. These data provide evidence that cancer cells modulate their metastatic niche via the upregulation of SMLR1 and physical interaction with immunosuppressive macrophages in the liver.

## 1. Introduction

Despite decades of research on drug and treatment development, improvements in metastatic colon cancer therapies have been limited [1]. The liver is the most frequent metastatic site for CRC, and CRLM is associated with poor disease outcomes [2]. CRC cells can undergo metabolic and epigenetic reprogramming to adapt their growth in their metastatic niche [3–5]. These findings suggest potential new strategies for targeting CRLM specifically via such reprogramming mechanisms and their interaction with the liver microenvironment.

Cancer immunotherapy, including immune checkpoint blockade (ICB), is a revolutionary treatment for cancer [6]. In addition to eradicating the primary tumor, cases of metastatic cancer regression have been documented in multiple clinical trials [7]. However, a large proportion of CRC patients showed no or minimal responses to ICB, and systemic resistance to ICB is common among patients with liver metastasis [8]. Emerging evidence suggests that, aside from CRC mutation burden, the hepatic microenvironment may contribute to the lack of ICB efficacy against CRLM, such as the existence of FasL^+^CD11b^+^F4/80^+^ macrophages that trigger systemic Fas^+^CD8^+^ T cell apoptosis [9]. Therefore, understanding the interaction between metastatic CRC cells and immune cells in their microenvironment may provide new insights to overcome CRLM resistance to immunotherapies.

In this study, we found small leucine-rich protein 1 (SMLR1) is highly expressed in CRLM samples. The over-expression of SMLR1 upon metastasizing to the liver is universal to cancers originating from different lineages. Using proteomics and cell retention assay, we demonstrated that SMLR1 on the tumor cell membrane facilitates physical interaction between the cancer cells and macrophages expressing mannose receptor C-type 1 (MRC1) and sialic acid binding Ig-like lectin 1 (SIGLEC1). Therefore, we unveiled an immune modulatory axis that is governed by cancer cell-intrinsic ectopic expression of SMLR1.

## 2. Results

### 2.1 Cancer cells that metastasize to the liver up-regulate SMLR1

Transcriptomics profiles between CRLM and primary tumor tissues were compared (Figure S1A). Among 3 independent cohorts, each of which comprised at least 10 CRLM samples, 349 genes were found to be highly differentially expressed, and 49 genes were consensually enriched in CRLM tissues. While CRLM up-regulated genes that encode proteins localize to extracellular spaces, mitochondrial matrix, and membranes, we focused on 13 membrane protein-encoding genes due to their potential roles in the tumor microenvironment crosstalk (Figure S1B). From them, we further extracted the putative CRC-specific genes that are upregulated in primary CRC when compared to the adjacent normal tissues (Figure S1C). This stepwise screening resulted in 2 candidates, *SMLR1* and hepcidin antimicrobial peptide (*HAMP*) (Figures 1A and S1C). While *HAMP* has been found to be a negative prognosis marker for CRC patients [10], the emergence of *SLMR1* was rather intriguing as there had been no publication reporting any SMLR1 function at the time of our investigation, and till now only one recent report linking SMLR1 with VLDL secretion and hepatosteatosis [11]. We also searched the protein atlas for the protein expression of SMLR1 and found strong membranous staining of SMLR1 among CRC tissues (Figure S1D).

The enhanced *SMLR1* mRNA expression is specific to CRLM as CRC peritoneal metastases displayed no changes in *SMLR1* expression levels compared to primary tumors (Figure 1B). In a meta-analysis of publicly available liver metastasis datasets, cancers originating from other organs also displayed an augmented *SMLR1* expression upon liver metastasis (Figure 1C), but not metastasis in other organs (Figure 1D). Two additional independent cohorts employing microdissection during sample preparation confirmed the high *SMLR1* expression among the liver metastasis cancers (Figure 1E). We further examined *SMLR1* expression by qPCR in 2 pairs of matched CRC organoids that were previously characterized and reported [4], in which organoids derived from liver metastasis expressed higher *SMLR1* when compared with the matched cancer organoids derived from the primary tumor (Figure 1F).

### 2.2 High SMLR1 expression by the cancer is associated with poor prognosis

Survival data from TCGA further indicates that CRC patients with higher *SMLR1* expression experienced a significant reduction in disease-free survival (Figure 2A). We then employed an established *in vivo* CRLM model via splenic injection to test the potential impact of SMLR1 expression on liver metastases (Figure 2B). Among a panel of colorectal cancer cell lines, CT26 and CRC57 had low basal *SMLR1* expression levels in culture while CACO2 had the highest *SMLR1* expression level (Figures S2A–S2C). We also validated the high and membranous protein expression in CACO2 cells with immunocytochemistry (Figure S2D). In a synergic mouse model, mice injected with CT26 cells with ectopic expression of SMLR1 (SMLR1-OE) had shorter lifespans (Figure 2C). The SMLR1-OE mice displayed high liver metastasis burden as shown by the IVIS images on day 10 (Figure 2D). In an independent non-survival cohort using engineered patient-derived CRC57 cells and NSG mice, SMLR1 expression was associated with liver metastasis but not with the primary tumor size (Figure 2E). We observed similar results in a tail vein injection model, in which a significantly higher liver metastasis burden was found among mice injected with SMLR1-OE cancer cells (Figure S2E). CACO2 cells carrying either the control vector or SMLR1 knockdown (*SMLR1*-gRNA expressing) vectors did not lead to significant CRLM, are commonly used as an *in vitro* normal colon epithelial barrier model (Figure S2F). Altogether, SMLR1 expression in CRC cells seemed to promote cancer cell survival in the liver niche upon metastasis with unknown mechanisms.

### 2.3 SMLR1 is associated with MRC1 and SIGLEC1

While a recent study demonstrated SMLR1’s role in lipid metabolism, its function in cancer remains elusive [11]. We constructed a correlation network between *SMLR1* mRNA expression and the whole tissue transcriptome. While a strong correlation could be found between genes involved in fatty acid, amino acids, and drug metabolisms in normal liver tissues, connections between *SMLR1* and lipid metabolism genes were less obvious among cancerous tissues (Figure S3A). Furthermore, complement and coagulation cascades and cytokine-cytokine receptor interaction genes were enriched in CRLM tissues (Figure S3A). Consistent with SMLR1 being a membrane-bound protein, we observed a correlation between *SMLR1* and genes on the external side of the cell membrane in the CRLM samples (Figure S3B).

As SMLR1 is a membrane protein, we used proteomics to identify intra- and intercellular protein-protein interactions respectively after pulling down tagged SMLR1 from metastatic liver tissue harvested from the xenogeneic cancer cell injection model (Figure 3A and Table S1). Consistent with the gene expression network analysis, we found that the SMLR1 protein was associated with human proteins localized in the mitochondria membrane and complexes on the plasma membrane (Figures 3B and S3). Notably, the SMLR1-associated mouse proteins, which indicate potential interaction between SMLR1 and the hepatic stromal environment, showed a bias towards innate immune cells such as macrophages (Figure 3B). Western blot of the pull-down SMLR1 confirmed that SMLR1 binds with Siglec1 and Mrc1, which are frequently found in specialist antigen-presenting cells (Figure 3C). Moreover, SMLR1 also interacted with the human homolog of SIGLEC1 and MRC1 (Figure 3D). Compared to mice injected with control vector expressing cancer cells, perfused liver from mice injected with SMLR1-OE cancer cells, which bore noticeable metastatic tumors, have elevated Mrc1^+^ and Siglec1^+^ cell populations, especially the Siglec1^+^ population (Figures 3E and S4). Furthermore, these mice had higher Siglec1^+^Mrc1^+^ cells in the perfused liver compared with control mice (Figure 3F).

### 2.4 MRC1+SIGLEC1+ macrophages are immunosuppressive

Because the Siglec1^+^Mrc1^+^ cell is a population of liver resident cells that is under-characterized, we first analyzed a published single-cell transcriptomic data from colon cancer liver metastasis together with its adjacent normal liver tissues [12]. We found that while cells expressing high *MRC1* spread across both dendritic cells and macrophages, cells simultaneously expressing high *MRC1* and *SIGLEC1* were only concentrated in both niche-associated and malignancy-associated macrophage subpopulations (Figure 4A). Compared to normal liver tissue, the liver metastasis tissue microenvironment harbored a significantly higher proportion of *CD3E*^+^, *FCN1*^+^, and *SPP1*^+^ macrophages co-expressing *MRC1* and *SIGLEC1* (Figure S5A). Overall, *MRC1*^+^ and *SIGLEC1*^+^ cells in the liver tumor microenvironment were enriched with signatures of differentiated macrophages in culture (Figures 4B and S5B). In addition, these double-positive cells were also enriched with M2 macrophage signatures when compared with cells that expressed either *SIGLEC1* or *MRC1* (Figure 4C). Within the macrophage population, we observed that *MRC1*^+^*SIGLEC1*^+^ cells were upregulated with multiple myeloid checkpoints and inhibitory receptors [13] (Figure 4D). Notably, triggering receptor expressed on myeloid cells 2 (*TREM2*), a membrane protein that plays an immunosuppressive role in cancer, was only co-expressed in *MRC1*^+^*SIGLEC1*^+^ cells rather than cells expressed in either *MRC1* or *SIGLEC1* alone.

### 2.5 SMLR1 allows cancer cells to physically bind to macrophages

As a membrane-bound protein, we hypothesized that SMLR1 potentially functions as an anchor protein for physical interaction with macrophages. *SMLR1*-expressed cancer cells are significantly more likely to be spatially associated with macrophages expressing both *SIGLEC1* and *MRC1* according to the spatial transcriptomics profile [14] (Figures 5A and 5B). This colocalization relationship between these two cell populations was weakened after neoadjuvant treatment (Figures S6A–S6C). We then performed the cell retention assay to verify CRC and macrophage interaction via SMLR1 (Figure S6D). Expression of SMLR1 in CRC57 cells enhanced its interaction with IL-4-induced bone marrow macrophage expressing both Siglec1 and Mrc1 (Figures 5C and S6E). Ectopic expression of SMLR1 led to the same effects in MC38 cells that had low basal SMLR1 expression levels (Figure 5D). On the other hand, in CACO2 cells that had high basal SMLR1 expression levels, knockdown of SMLR1 by guide-RNA abrogated the interaction between CACO2 and macrophages (Figure 5E). These data suggested a pivotal rule of SMLR1 in the physical interaction between cancer and tumor-associated macrophages.

## 3. Discussion

Seeding and subsequent adaptation to the liver are major obstacles that metastatic cancer cells need to overcome [15]. Cell rewiring is one of the ways for cancer cells to survive in the new niche [16]. The function of SMLR1 in cancer was unknown although one recent study reported a role of SMLR1 in VLDL trafficking and steatosis [11]. Our study suggests that SMLR1 expression is associated with CRLM and may play a role in modulating the immune microenvironment of the liver metastatic niche. While cancer cells can recruit pro-tumorigenic macrophages via cytokines and chemokines [17,18], SMLR1 seems to be a tissue-specific surface protein that allows metastatic cancer cells to interact with TAM directly in the liver.

Liver metastases are known to be immunosuppressive compared to other metastatic sites. We showed that SMLR1 expression facilitated the binding of cancer cells to immunosuppressive macrophages. MRC1 (CD206) is a well-established marker for M2 macrophages supporting cancer survival and metastatic spreads [19]. The acquired physical interaction with MRC1 macrophages by over-expressing SMLR1 might synergize the EGF-signaling loop between cancer cells and macrophages, as it has been shown that MRC1^+^ cells produce EGF in a paracrine manner, thus fueling the development of cancer [20]. Nevertheless, controversial findings have been reported on SIGEC1-positive (CD169^+^) macrophages. For instance, high expression of SIGLEC1 was associated with poor prognosis in breast cancer patients [21], but SIGLEC1^+^ macrophages showed antitumor activity in glioblastoma [22]. We found that only *SIGLEC1* and *MRC1* co-expressing macrophages expressed high levels of inhibitory receptors. Therefore, the role of SIGLEC1 cells may be subpopulation specific. Targeting this macrophage subpopulation rather than the whole SIGLEC1^+^ population might lead to more favorable clinical outcomes [23]. Moreover, future MRC1 and SIGLEC1 double knockout models would aid the exploration of the complex crosstalk between cancer cells and macrophages and the signal cascades underneath.

## 4. Methods

### 4.1 Cell lines

CACO2, SW620, LS174T, HT29, HCT116, DLD1, CT26, and MC38 cells were purchased from ATCC. Patient-derived colorectal cancer cell lines CRC57, CCSC, CRC12X, CRC247, and CRC119 were a gift from David Hsu’s lab at Duke University. Colorectal cancer cell lines were maintained in RPMI1640 medium supplemented with 10% FBS and 1% penicillin/streptomycin cocktail (P/S). Organoids were cultured according to the previously published protocol [24]. Bone marrow macrophages were isolated from Balb/c mouse and maintained in DMEM supplemented with 10% FBS and 1 ng/mL macrophage colony-stimulating factor (M-CSF1). Macrophages were treated with 10 ng/mL IL4 for 24 hours. THP1 monocytes were maintained in RPMI-1640 medium supplemented with 10% FBS and 0.05 mM 2-mercaptoethanol.

### 4.2 Cell engineering

In SMLR1 over-expression, SMLR1-flag cDNA (Genescript, clone OHu10377) was cloned to pHIV-eGFP (Plasmid #21373) empty vector while the pHIV-eGFP vector served as control. Lentivirus transduction of these constructs was performed to obtain stable expression cells. These cells were further purified and selected by puromycin and cell sorting gated with eGFP expression. To facilitate *in vivo* imaging, eGFP-labeled cells were transduced with another plasmid expressing mcherry and luciferase. These secondary transduced cells were selected via cell sorting fated with the expression of eGFP and mcherry. For guide RNA (gRNA) knockdown of SMLR1, gRNA expressing plasmid were transduced to the cells. Two gRNA constructs targeting *SMLR1* were TGATCGCCAACATAATCCCA and AATCAGTTTGATCCTGAAGT (Genescript). Cells were first selected with puromycin. Cells were then transduced again with a plasmid expressing mcherry and luciferase. The engineered cells were purified by cell sorting gated with the expression of mcherry.

### 4.3 Animals

Balb/c mice were ordered from JAX and NSG mice were purchased from Duke Rodent Gnotobiotic and Breeding Core. Male and Female mice aged 6 – 10 were used for experiments. For the spleen injection models, splenectomy was performed on day 2 – day 5 depending on the average signal of the primary tumor in the same cohort. For the survival cohort, 5×10^5^ engineered CRC57 cells were injected into the spleen on day 0. The survivorship of the mice was monitored till day 21. On day 14, *in vivo* imaging (IVIS) was performed by intraperitoneally injecting 150 mg/kg luciferase into each mouse. For the non-survival cohort, 5×10^5^ engineered CRC57 cells were injected into the spleen on day 0. the mice were euthanized on day 14 for ex vivo liver imaging. For the proteomic cohort, 5×10^5^ engineered CRC57 cells that expressed SMLR1-OE were injected into the spleen on day 0, then euthanized on day 14. To quantify liver resident Siglec1^+^Mrc1^+^, mice were either injected with 5×10^5^ engineered CRC57 cells, and mice were euthanized on days 12–14. For tail vein injection models, 5×10^5^ engineered cells were injected, and mice were monitored for up to 3 weeks.

### 4.4 Analysis of public datasets

The Cancer Genome Atlas Program (TCGA) data (primary tumor and adjacent normal tissues) were downloaded from Xena [25]. Transcriptomic count data were normalized and processed by DESeq2. Publicly available manually curated TCGA survival data were used. Healthy colon tissue (sigmoid) mRNA expression data were obtained from the GTEx portal and normalized in DESeq2. Gene biweight midcorrelation (bicor) matrixes were calculated using the package WGCNA [26]. The bicors between *SMLR1* and all other expressed genes were used for gene set enrichment analysis against the KEGG database and gene ontology database (cellular compartment). Human cancer cell lines’ SMLR1 expression data were obtained by Cancer Cell Line Encyclopedia (CCLE) [27] and Tumor Immune Syngeneic MOuse (TISMO) provided the *SMLR1* expression information for mouse cancer cell lines [28]. Cancer with liver metastasis data was mined from GEO [29–40]. Only studies profiling at least 10 metastasis samples were included. RNAseq datasets were analyzed by DESeq2, and microarray datasets were analyzed by affy and beadarray according to the chip design before conducting differential gene analysis by limma. Immunohistochemistry staining images from the protein atlas were accessed at https://www.proteinatlas.org/ENSG00000256162-SMLR1/pathology/colorectal+cancer#img [41].

### 4.5 Proteomics and data analysis

Colon cancer liver metastasis tumor tissues were harvested from mice and dissociated by the tumor dissociation kit (Miltenyi Biotech cat# 130-095-929). Proteins were extracted by RIPA lysis buffer (Thermofisher cat# 89901). Pull-down of SMLR1-flag was performed by using the Anti-FLAG^®^ M2 Magnetic Beads (Sigma cat# M8823). For in-solution analysis, samples were supplemented with 5% SDS, reduced for 15 min at 80C, alkylated with 20 mM iodoacetamide for 30 min at room temperature, then supplemented with a final concentration of 1.2% phosphoric acid and 609 μL of S-Trap (Protifi) binding buffer (90% MeOH/100mM TEAB). Proteins were trapped on the S-Trap micro cartridge, digested using 20 ng/μL sequencing grade trypsin (Promega) for 1 hour at 47 °C, and eluted using 50 mM TEAB, followed by 0.2% FA, and lastly using 50% ACN/0.2% FA. All samples were then lyophilized to dryness.

Each sample was subjected to chromatographic separation on a Waters MClass UPLC equipped with a 1.7 μm HSS T3 C18 75 μm I.D. X 250 mm reversed-phase column (NanoFlow data). The mobile phase consisted of (A) 0.1% formic acid in water and (B) 0.1% formic acid in acetonitrile. 3 μL was injected and peptides were trapped for 3 min on a 5 μm Symmetry C18 180 μm I.D. X 20 mm column at 5 μl/min in 99.9% A. The analytical column was then switched in-line and a linear elution gradient of 5% B to 40% B was performed over 90 min at 400 nL/min. The analytical column was connected to a Fusion Lumos mass spectrometer (Thermo) through an electrospray interface operating in a data-dependent mode of acquisition. The instrument was set to acquire a precursor MS scan from m/z 375–1500 at R = 120,000 (target AGC 2e5, max IT 50 ms) with MS/MS spectra acquired in the ion trap (target AGC 1e4, max IT 50 ms). For all experiments, HCD energy settings were 30% and a 20 s dynamic exclusion was employed for previously fragmented precursor ions.

Raw LC-MS/MS data files were processed in Proteome Discoverer 2.5 (Thermo Scientific) and then submitted to independent Sequest database searches against a Human and Mouse protein database containing both forward and reverse entries of each protein. Search tolerances were 2 ppm for precursor ions and 0.8 Da for product ions using trypsin specificity with up to two missed cleavages. Carbamidomethylation (+57.0214 Da on C) was set as a fixed modification, whereas oxidation (+15.9949 Da on M) was considered a dynamic mass modification. Data were then imported into Scaffold (v5.0) and scoring thresholds were adjusted to a maximum 1% false discovery rate.

Identified proteins that do not contain shared peptides were retained for further analysis. Proteins found in controls were excluded to shortlist proteins associated with SMLR1. Human and mouse proteins were analyzed separately by over-representation test searched against the cellular compartment Gene Ontology database and CellMarker database [42].

### 4.6 Western Blot

An independent pull-down assay was performed on colon cancer liver metastasis tissue samples from mice inoculated with SMLR1-OE-flag CRC57 cells. 1:1000 α-SMLR1 (Abcam cat# ab204982), 1:1000 α-Siglec-1 (abcam cat# ab53443), and 1:1000 α-Mrc1 (Abcam cat# 64693) were used as primary antibodies. To assess the interaction between SMLR1 and human homologs, SMLR1-OE-flag CRC57 cells were co-cultured with THP1 cells before the pull-down assay. 1:1000 α-SIGLEC-1 (Abcam cat# 245742) and 1:1000 α-MRC1 (Abcam cat# 252921) were used as primary antibodies.

### 4.7 Real-time PCR

RNA was extracted by Quick-RNA Midiprep Kit (Zymo Research R1056) and reverse transcribed by High-Capacity cDNA Reverse Transcription Kit (Applied Biosystems 4368814). Taqman probes, *SMLR1*: Hs00400907_m1; *ACTIN*: Hs99999903_m1; *Cd169*: Mm00488332_m1; *Cd206*: Mm01329359_m1; *Actin*: Mm00607939_s1, were ordered from Thermofisher Scientific. Gene expression was normalized to actin expression.

### 4.8 Flow cytometry

Liver perfusions were performed after euthanasia by carbon dioxide inhalation. In brief, mouse liver was perfused with pre-warmed perfusion buffer I (500 ml sterile phosphate-buffered saline (PBS), 5 ml buffer A, 2.5 mL 1 M glucose (sterile filtered), 0.5 mL 200 mM EDTA) for 10 minutes and per-warmed buffer II (500 mL sterile PBS, 5 mL buffer A, 10 mL 1 M HEPES, 2.5 mL 1 M glucose (sterile filtered), 1 mL 500 mM CaCl2. pH adjusted to 7.4 with 2 M NaOH) with 3600U of collagenase type IV (Sigma). The homogenized liver lysate was collected in a 50 mL tube and hepatocytes were removed by centrifugation at 58 × g for 2 minutes at 4 °C for 3 times. The supernatant was finally pelleted by centrifugation at 300 × g for 10 minutes at 4 °C. Red blood cells were removed, and cells were blocked with 2% BSA and 1:200 TruStain FcX^™^ PLUS (Biolegend cat# 156603). After resuspending cells in sorting buffer (1x PBS, 2.5mM EDTA, 25mM HEPES, PH7.0, 1% FBS, 1% Pen-Strep, 100U/ml Dnase I), cells were stained with α-Cd206 (BioLegend cat# 141716), α-Cd169 (BD Biosciences cat# 566604) for 30 minutes on ice. Cells were analyzed by Sony Sorter SH800. For retention assay, mCherry-expressing cancer cells were counted.

### 4.9 Single-cell transcriptomic analysis and pathway analysis

Processed single-cell transcriptomic data were downloaded from GEO [12], with accession number GSE164522. Based on the cell clustering results originally proposed by the authors, cells were further characterized by the expression of *MRC1* and *SIGLEC1*. Analysis was focused on macrophage (mph), monocyte (mono), and dendritic cells (DC) in colon cancer liver metastasis (MT) and adjacent normal liver tissue (MN). Gene set enrichment analysis (GSEA) was searched against the immunologic signature gene set in the Human Molecular Signatures Database (MSigDB). Single sample GSEA (ssGSEA) score was calculated for each single cell in MT and MN samples and the differential gene set enrichment among cell groups was calculated with limma. Differential gene expression among different cell groups was performed using the FindMarker function in Seurat which relies on the Wilcoxon Rank Sum test.

### 4.10 Spatial transcriptomic analysis and local colocation quotient quantification

Processed spatial transcriptomic data were obtained from the published study [14]. Data were further processed by Seurat. Cancer and its associated stromal regions were visually labeled by overlaying clustered spots and the accompanied H&E-stained slide. Colocalizations between SMLR1^+^ cancer cells and TAM were estimated by the local colocation quotient (LCQ) [43]. Spots that were classified as normal liver were excluded from the analysis. Spots that were located in the cancerous region and expressed *SMLR1* were defined as spots that contained *SMLR1*^+^ cells. Macrophages in cancerous spots and adjacent spots directly in contact with the cancerous region were defined by the expression of *CD68* and *MRC1* and/ or *SIGLEC1*. LCQ was defined as:

$$LCQ=\frac{\frac{n_{b}}{n_{a}}}{\frac{N_{b}-1}{N_{a}-1}}$$

where *N*_*a*_ is the total number of *SMLR1*^+^ spots in the slide, *N*_*b*_ is the total number of spots of interest in the slide, *n*_*a*_ is the number of *SMLR1*^+^ spots within a 65 μm radius of the center *SMLR1*^+^ spot, and *n*_*b*_ is the number of spots of interest within a 65 μm radius of the center *SMLR1*^+^ spot. Null LCQs were generated by permutating the spot location. The mean LCQ for each tissue slide was deduced by averaging the LCQ of all *SMLR1*^+^ spots of the slide. The *p*-value was calculated by the permutation test with 1000 randomizations. The *p*-value indicated the probability of the random instances of *SMLR1*^+^ spots being colocalized with *MRC1* and/ or *SIGLEC1* spots to a higher degree than the observed actual data in a permutation test that randomized the spot location. Therefore, a low *p*-value indicated a low chance that the spatial association between *SMLR1* and *MRC1/SIGLEC1* was random.

### 4.11 Retention assay

The assay is adapted from a published protocol [44]. Cultured cancer cells and macrophages were gently dissociated by trypsin. 1 × 10^5^ macrophages in PBS were seeded on Poly-D-Lysine coated 24-well plate (Corning 356414) and incubated on ice for 5 minutes. The remaining attachment sites were blocked by adding 2% bovine serum albumin (BSA) in PBS. 1 × 10^5^ cancer cells were then added to each well and incubated on ice for 510 minutes. After two washes with cold PBS, retained cells were resuspended in 100 μl of PBS by agitating the plate. Cancer cells were counted by flow cytometry.

### 4.12 Statistical Analysis

All data were presented as mean ± SEM. Statistical analyses were performed in R. Comparisons between two groups were performed by the two-sided student t-test unless otherwise specified. When more than two groups were compared, the post hoc Tukey multiple comparison correction was performed after analysis of variance (ANOVA). Unless specified otherwise, asterisks in the figures indicate statistical significance *: *p* < 0.05, **: *p* < 0.01, ***: *p* < 0.001, ****: *p* < 0.0001.

## Supplementary Material

Figure S1-S6 (CHP2401010003)

Table S1 (CHP2401010003)

The following supplementary materials are available on the website of this paper:

1. Figure S1: Enhanced expression of membranous proteins among CRC tissues.
2. Figure S2: *SMLR1* expression in colon cancer models.
3. Figure S3: Gene set enrichment analysis of genes correlated with *SMLR1* mRNA expression.
4. Figure S4: Gating strategy of liver resident cells.
5. Figure S5: Characterization of *SIGLEC1*^+^*MRC1*^+^ cells in CRLM tissues.
6. Figure S6: Cancer cell-macrophage interaction among patients and *in vitro* models.
7. Table S1: List of proteins associated with SMLR1 from Proteomics.

## Figures and Tables

**Figure 1:**
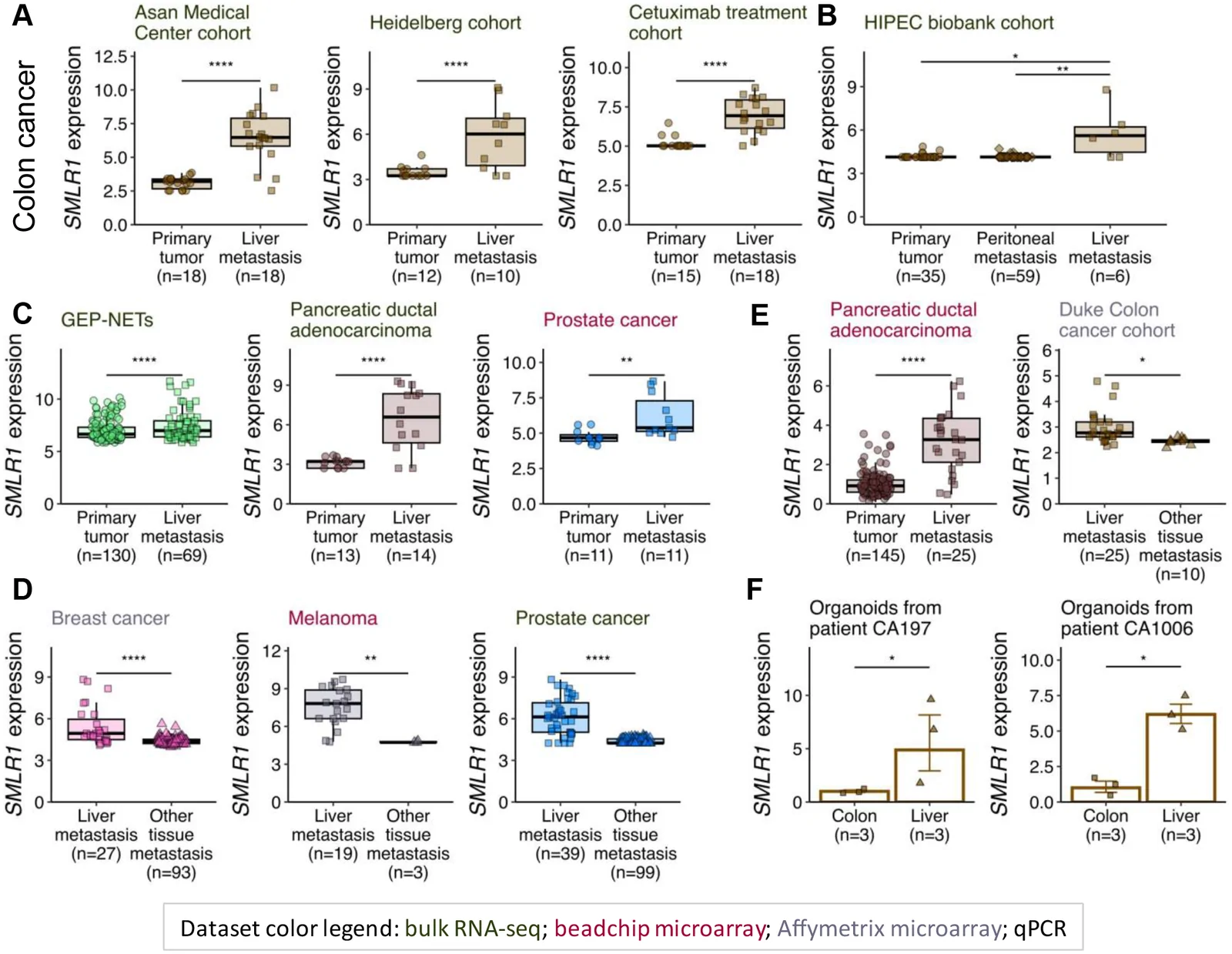
SMLR1 over-expression among cancer liver metastases. (**A**) *SMLR1* gene expression in primary colon cancer tumor and metastatic tumor in three independent cohorts (GSE50760, GSE145429, and GSE183984). (**B**) Comparison of *SMLR1* gene expression among primary colon cancer tumor, peritoneal metastasis, and liver metastasis (GSE190609). (**C**) *SMLR1* gene expression in primary tumor and liver metastasis among cancer originated from a variety of tissues (GSE98894, GSE151580, and GSE74367). (**D**) *SMLR1* gene expression in the liver metastasis and metastatic tumor in other tissues (GSE22154, GSE147250, and GSE56493). (**E**) Quantification of *SMLR1* gene expression on tissues after micro-dissection among two cohorts (GSE71729 and GSE18549). (**F**) *SMLR1* mRNA expression quantified by qPCR in matched cancer organoids from two patients. GEP-NETs: gastroenteropancreatic neuroendocrine tumors; the number of samples in each group was included in the bracket on the x-axis. Data information: For box-plot: center line, median; box limits, upper and lower quartiles; whiskers, 1.5 x interquartile range. For bar-chart: data are presented as mean ± SEM. *p-value < 0.05, **p-value < 0.01, and ****p-value < 0.0001. Bulk RNA-seq data were analyzed by DESeq2 and data after variance stabilizing transformation were shown; beadchip microarray data were analyzed by limma and background-corrected quantile normalized data were shown; Affymetrix microarray data were analyzed by affy/ limma and robust multichip average data were shown; t-test were used for the significant inference for qPCR data and fold-change data normalized to colon samples were shown.

**Figure 2:**
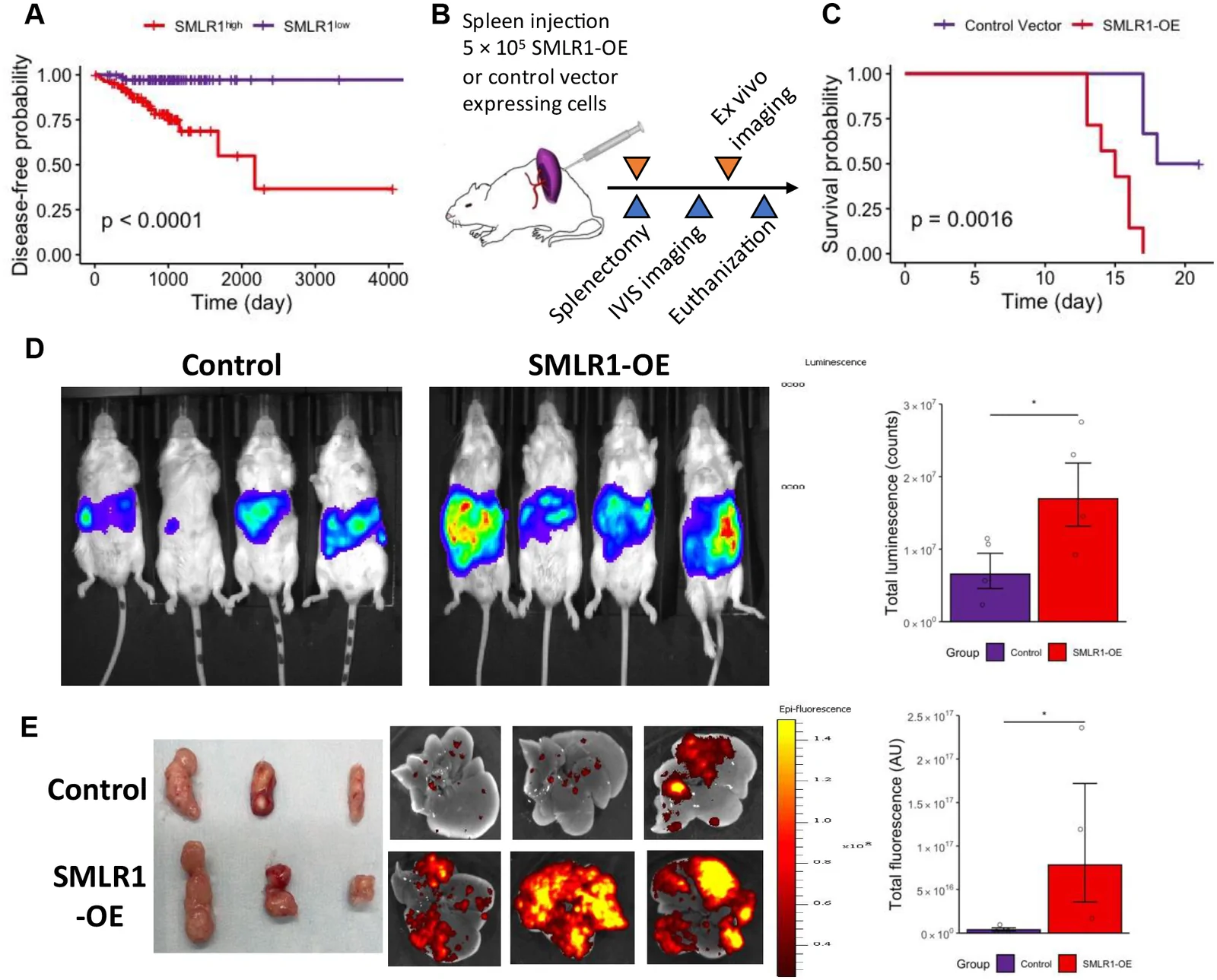
Effects of high SMLR1 expression in vivo. (**A**) Kaplan-Meier curve of relapse-free intervals among patients with high and low *SMLR1* expression. (**B**) Schematic diagram illustrating the survival (blue arrows) and non-survival (orange arrows) animal cohorts (n = 6). (**C**) Survival Curve for mice injected with either CT26 cells engineered with control or SMLR1-overexpression (SMLR1-OE) vector. (**D**) Representative luminescent images of mouse abdomen after luciferase injection. (**E**) Representative primary tumor photos (left) and metastatic liver cancer fluorescent images (right) among mice injected with either CRC57 cells engineered with control or SMLR1-overexpression (SMLR1-OE) vector. Data information: Data are presented as mean ± SEM. *p-value < 0.05.

**Figure 3:**
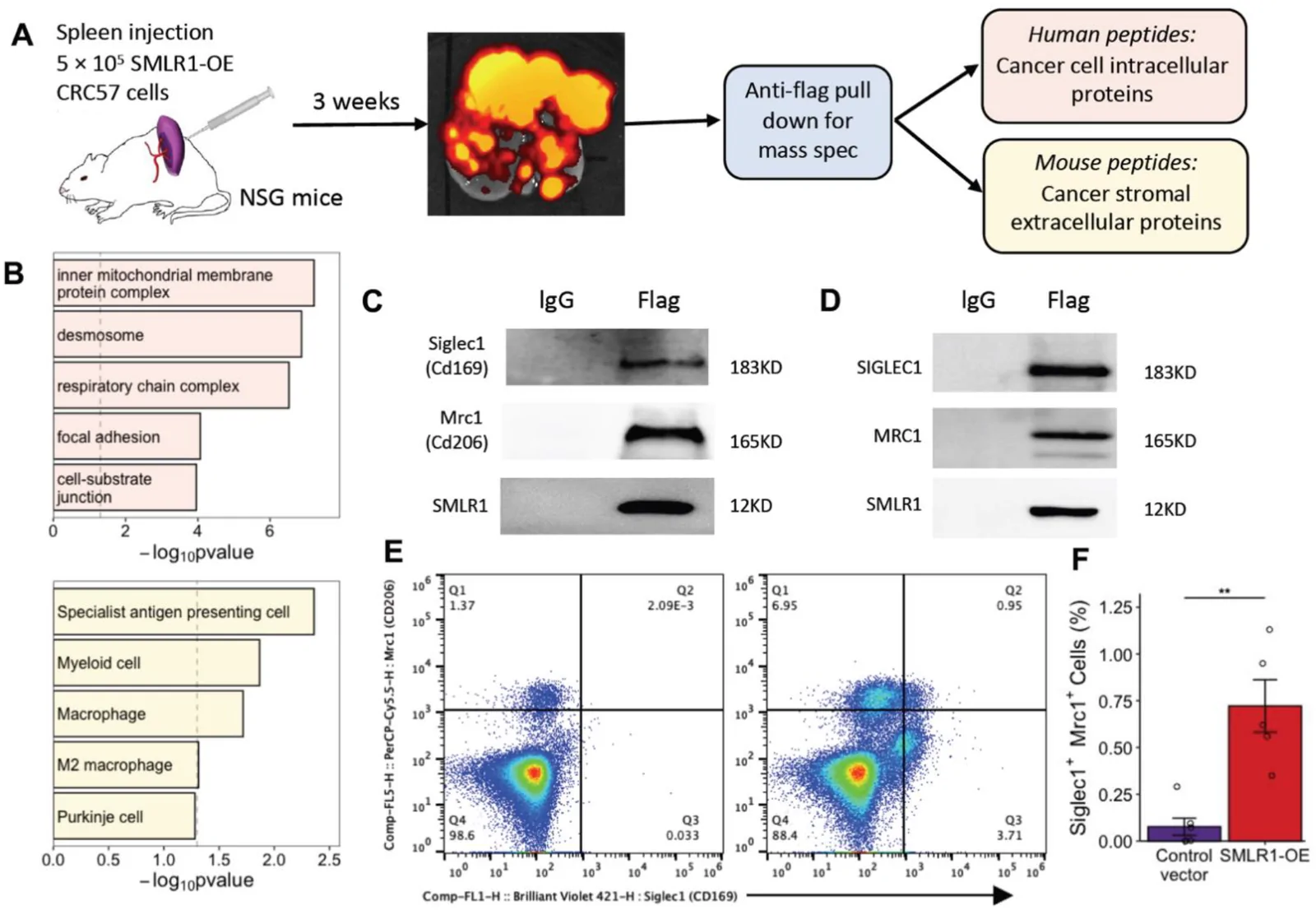
Association between cancer cell SMLR1 expression and stromal SIGLEC1 and MRC1 expression. (**A**) Schematic diagram showing the design of the experiment for discovering the binding partner of SMLR1 *in vivo*. (**B**) Over-represented pathways of human proteins enriched in FLAG pull-down sample (top) and over-represented cell types predicted from mouse proteins enriched in FLAG pull-down sample (bottom). (**C**) Western blots of pull-down assay of mouse protein from colon cancer liver metastasis tissue. (**D**) Western blots of pull-down assay of human proteins from CRC57 and THP cell co-culture. (**E**) Representative flow cytometry results showing cells that express Siglec1 and Mrc1 in perfused liver among mice injected with CT26 cells engineered with control vector (left) or SMLR1-OE (right). (**F**) Quantification of Siglec1+Mrc1+ cells in perfused liver among mice injected with CT26 cells engineered with control vector or SMLR1-OE (n = 5). Data information: Data are presented as mean ± SEM. **p-value < 0.01.

**Figure 4:**
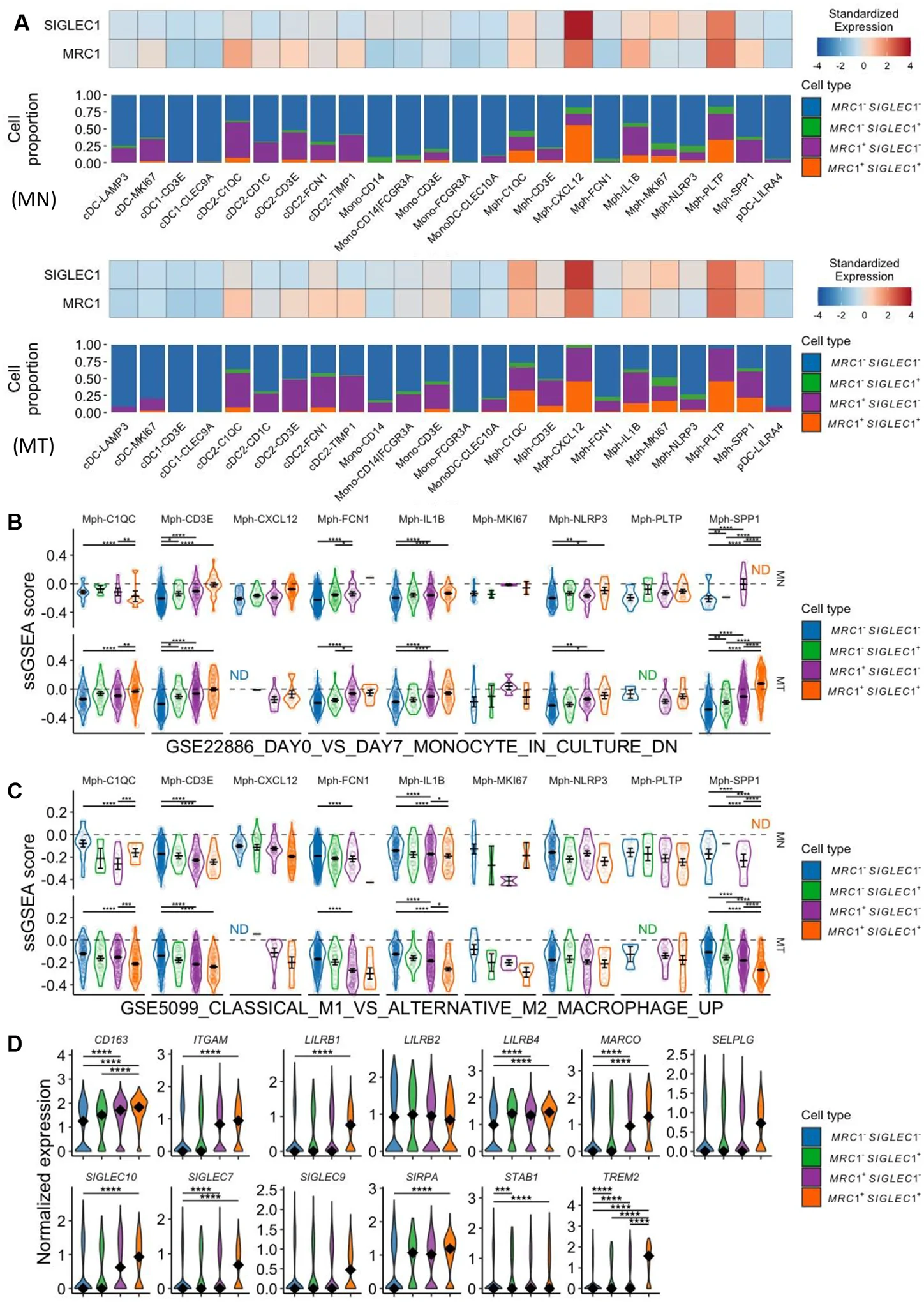
Characterization of SIGLEC1^+^MRC1^+^ cells. (**A**) Mean gene expression and proportion of cells that express *SIGLEC1* and *MRC1* among myeloid lineage in adjacent normal liver (MN, top panel) and liver metastasis of colon cancer (MT, bottom panel). (**B**) Single sample GSEA (ssGSEA) score of monocyte maturation signatures among cells grouped by cell clusters and *SIGLEC1* and *MRC1* expression status. (**C**) ssGSEA score of M1-polarized macrophage signatures among cells grouped by cell clusters and *SIGLEC1* and *MRC1* expression status. (**D**) Gene expression levels of myeloid checkpoints or repression receptors genes in macrophages grouped by *SIGLEC1* and *MRC1* expression. Data information: Data are presented as mean ± SEM. *p-value < 0.05, **p-value < 0.01, ***p-value < 0.001, and ****p-value < 0.0001. ND: not detected.

**Figure 5:**
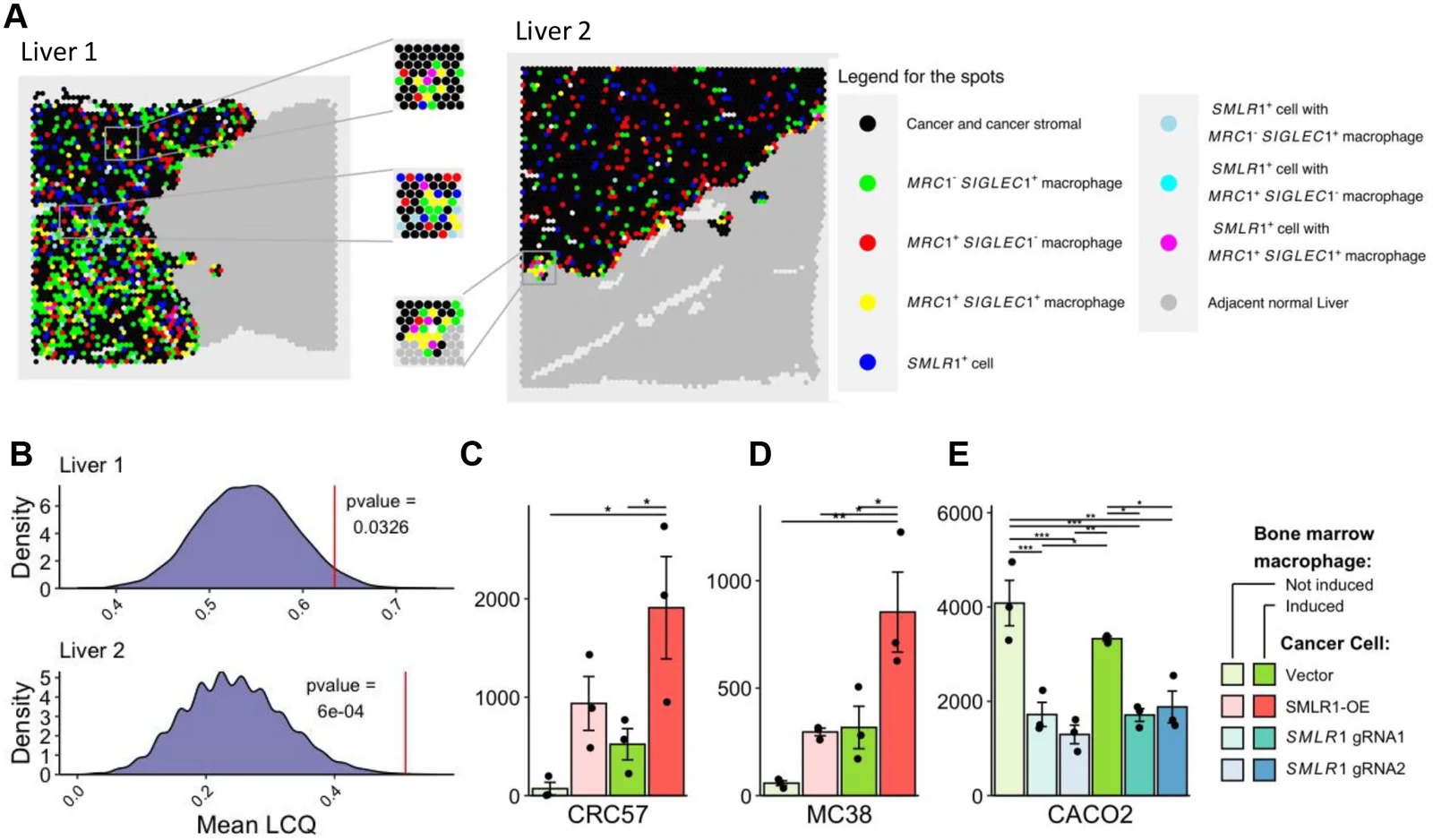
Interaction between SMLR1^+^ cancer cells and MRC1^+^SIGLEC1^+^ macrophages. (**A**) Spatial transcriptomics of colon cancer liver metastasis tissues from patients who were untreated before tumor resection. Left section: Liver 1; Right section: Liver 2. (**B**) Quantification of local colocation quotient (LCQ) between spots that contain *SMLR1*-expressing cancer cells and *SIGLEC1-MRC1* double positive macrophage. Simulated null mean LCQ were plotted as density plot and mean LCQ for Liver 1 (top) and Liver 2 (bottom) were indicated by the red vertical line. (**C**–**E**) Quantification of labeled cancer cells in retention assays (n = 3). Data information: Data are presented as mean ± SEM. *p-value < 0.05, **p-value < 0.01, and ***p-value < 0.001.

## Data Availability

The datasets generated during and/or analyzed during the current study are available from the corresponding authors upon reasonable request.

## Abbreviations

The following abbreviations are used in this manuscript:

CRLM: Colorectal cancer liver metastasis
CRC: Colorectal cancer
SMLR1: Small leucine-rich protein 1
ICB: Immune checkpoint blockade
MRC1: Macrophages expressing mannose receptor C-type 1
SIGLEC1: Sialic acid binding Ig-like lectin 1
HAMP: Hepcidin antimicrobial peptide
TCGA: The Cancer Genome Atlas
TREM2: Triggering receptor expressed on myeloid cells 2
VLDL: Very low density lipoprotein
