# Supplementary material for: Metastatic colorectal cancer cells upregulate SMLR1 that interacts with tumor-associated macrophages in the liver
Source: Cancer Heterog Plast. Author manuscript; Available in PMC 2026 Nov 5. (PMC13733557; doi:10.47248/chp2401010003)
Supplement: Supplementary file 1 [file NIHMS2217660-supplement-Figure_S1-S6__CHP2401010003_.pdf]

Cancer Heterogeneity and Plasticity
https://doi.org/10.47248/chp2401010003

Original Research

Metastatic colorectal cancer cells upregulate SMLR1 that interacts with tumor-associated macrophages in the liver

Chi Wut Wong 1,2,3,†,‡, Lihua Wang 3,†, Jorge Prado Balcazar 3, Yong Chen 3,4, Xiling Shen 1,5,‡,*

1. Terasaki Institute for Biomedical Innovation, Los Angeles, CA 90024, USA; Email: wwchi@mdanderson.org
2. Department of Pharmacology & Cancer Biology, Duke University School of Medicine, Durham, NC 27710, USA
3. Department of Biomedical Engineering, Pratt School of Engineering, Duke University, Durham, NC 27708, USA; Emails: ithacalihua@gmail.com (L.W.); Jprado.balcazar@gmail.com (J.P.B.)
4. Department of General Surgery, Xiangya Hospital, Central South University, Changsha 410013, China; Email: 178102111@csu.edu.cn
5. Department of Pathology, Duke University School of Medicine, Durham, NC 27710, USA

‡ Current Affiliation: GI Medical Oncology, UT MD Anderson Cancer Center, Houston, TX 77030, USA

† These authors contributed equally to this work.

* **Correspondence:** Xiling Shen; Email: xiling.shen@terasaki.org.

Supplementary Materials

Figure S1 Enhanced expression of membranous proteins among CRC tissues.
Figure S2 *SMLR1* expression in colon cancer models.
Figure S3 Gene set enrichment analysis of genes correlated with *SMLR1* mRNA expression.
Figure S4 Gating strategy of liver resident cells.
Figure S5 Characterization of SIGLEC1+MRC1+ cells in CRLM tissues.
Figure S6 Cancer cell-macrophage interaction among patients and in vitro models.

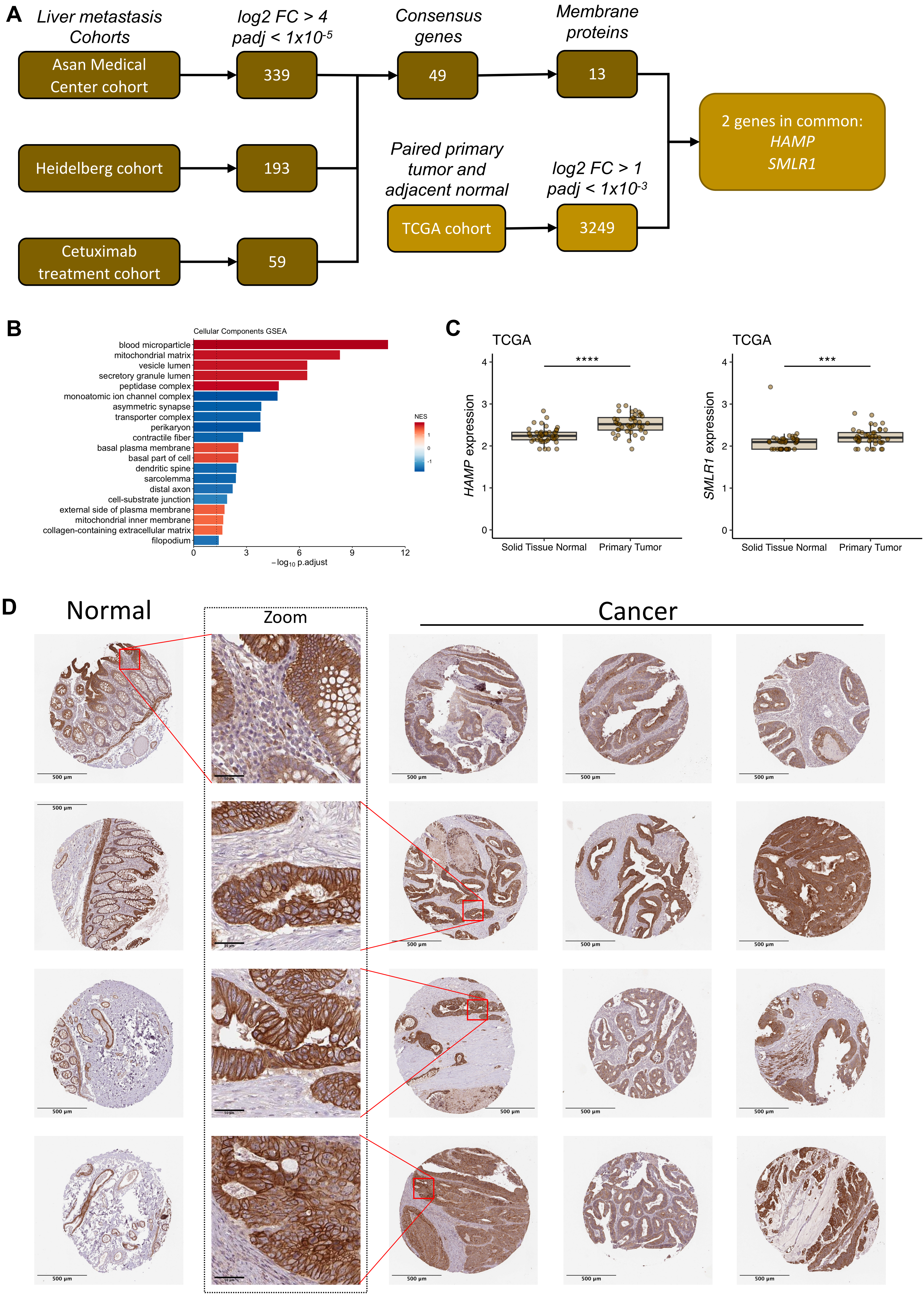
A
Liver metastasis Cohorts
log2 FC > 4 padj < 1x10-5
Consensus genes
Membrane proteins
Asan Medical Center cohort
339
49
13
Heidelberg cohort
193
Cetuximab treatment cohort
59
Paired primary tumor and adjacent normal
log2 FC > 1 padj < 1x10-3
TCGA cohort
3249
2 genes in common:
HAMP
SMLR1
B
Cellular Components GSEA
blood microparticle
mitochondrial matrix
vesicle lumen
secretory granule lumen
peptidase complex
monoatomic ion channel complex
asymmetric synapse
transporter complex
perikaryon
contractile fiber
basal plasma membrane
basal part of cell
dendritic spine
sarcolemma
distal axon
cell-substrate junction
external side of plasma membrane
mitochondrial inner membrane
collagen-containing extracellular matrix
filopodium
NES
−log10 p.adjust
C
TCGA
HAMP expression
****
SMLR1 expression
***
Solid Tissue Normal
Primary Tumor
D
Normal
Zoom
Cancer
500 µm

Figure S1 Enhanced expression of membranous proteins among CRC tissues

A Schematic diagram showing the gene candidate screening design and results.

B Gene set enrichment analysis of consensus genes against the cellular component (CC) gene ontology.

C Gene expression of *HAMP* (left) and *SMLR1* (right) in adjacent normal tissues and primary tumor among TCGA dataset.

D SMLR1 protein expression among human colon, rectum, and colorectal cancer tissue. Images credit: Human Protein Atlas (v23.proteinatlas.org).

Data information: For box-plot: center line, median; box limits, upper and lower quartiles; whiskers, 1.5x interquartile range. ***p-value < 0.001, and ****p-value < 0.0001.

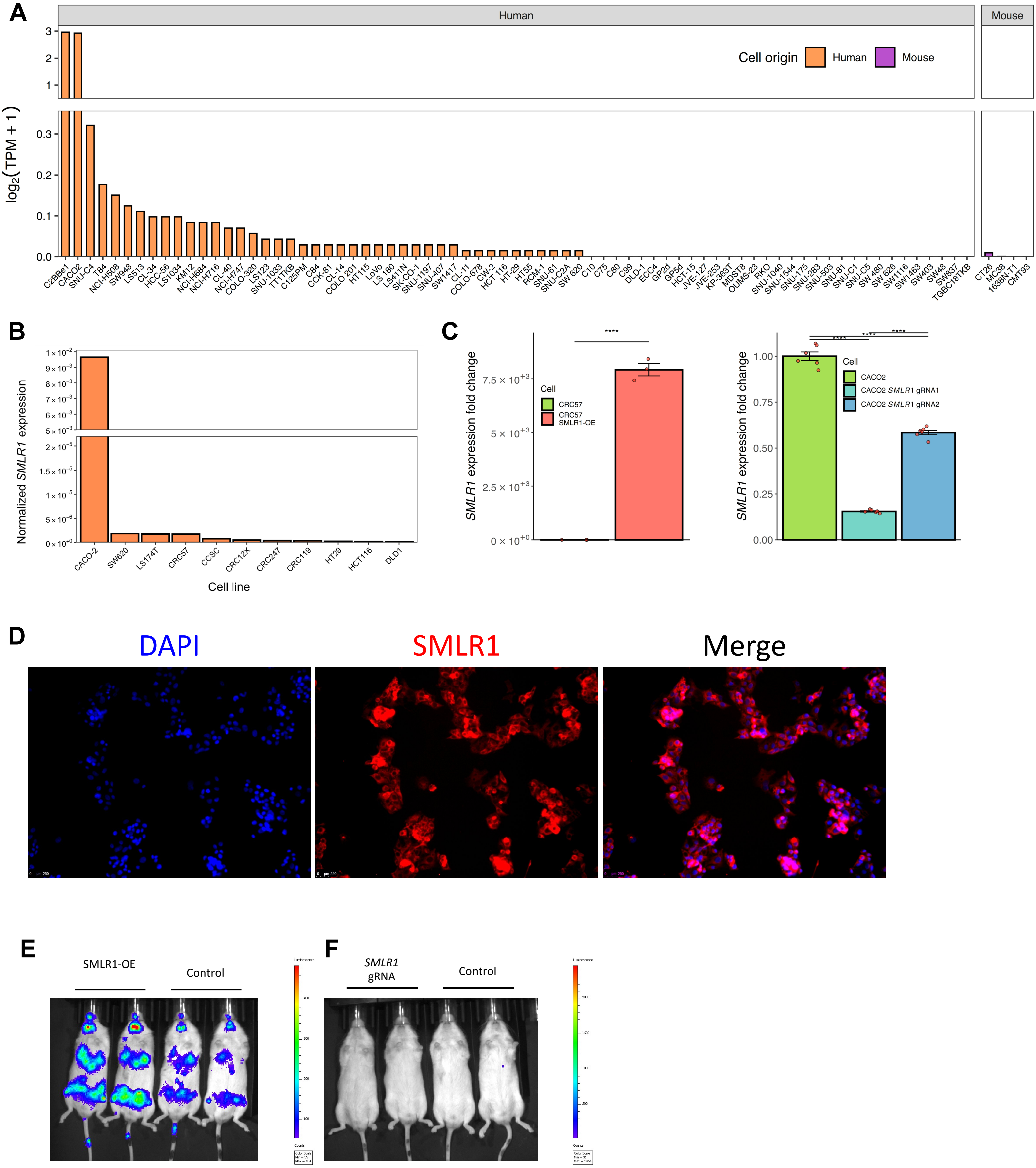
A
Human
Mouse
Cell origin
Human
Mouse
log2(TPM + 1)
B
Normalized SMLR1 expression
Cell line
CACO-2
SW620
LS174T
CRC57
CCSC
CRC12X
CRC247
CRC119
HT29
HCT116
DLD1
C
SMLR1 expression fold change
Cell
CRC57
CRC57 SMLR1-OE
CACO2
CACO2 SMLR1 gRNA1
CACO2 SMLR1 gRNA2
D
DAPI
SMLR1
Merge
E
SMLR1-OE
Control
F
SMLR1 gRNA
Control

Figure S2 *SMLR1* expression in colon cancer models

A *SMLR1* gene expression among human (from CCLE) and mouse (from TISMO) colon cancer cell lines.

B *SMLR1* gene expression in selected cancer cell lines derived from qPCR. *SMLR1* expression was normalized to *Actin* expression.

C *SMLR1* gene expression in engineered cancer cell lines derived from qPCR. *SMLR1* expression was normalized to *Actin* expression and data were shown as fold-change normalized to the vector controls.

D Representative image of SMLR1 protein expression in CACO2 cell in immunocytochemistry experiments.

E Representative luminescent images of NSG mice abdomen among mice tail vein injected with either SMLR1-OE or control vector expressing CRC57 for 1 week.

F Representative luminescent images of NSG mice abdomen among mice tail vein injected with either *SMLR1*-gRNA or control vector expressing CACO2 for 3 weeks.

Data information: For the bar chart: data are presented as mean ± SEM. ****p-value < 0.0001.

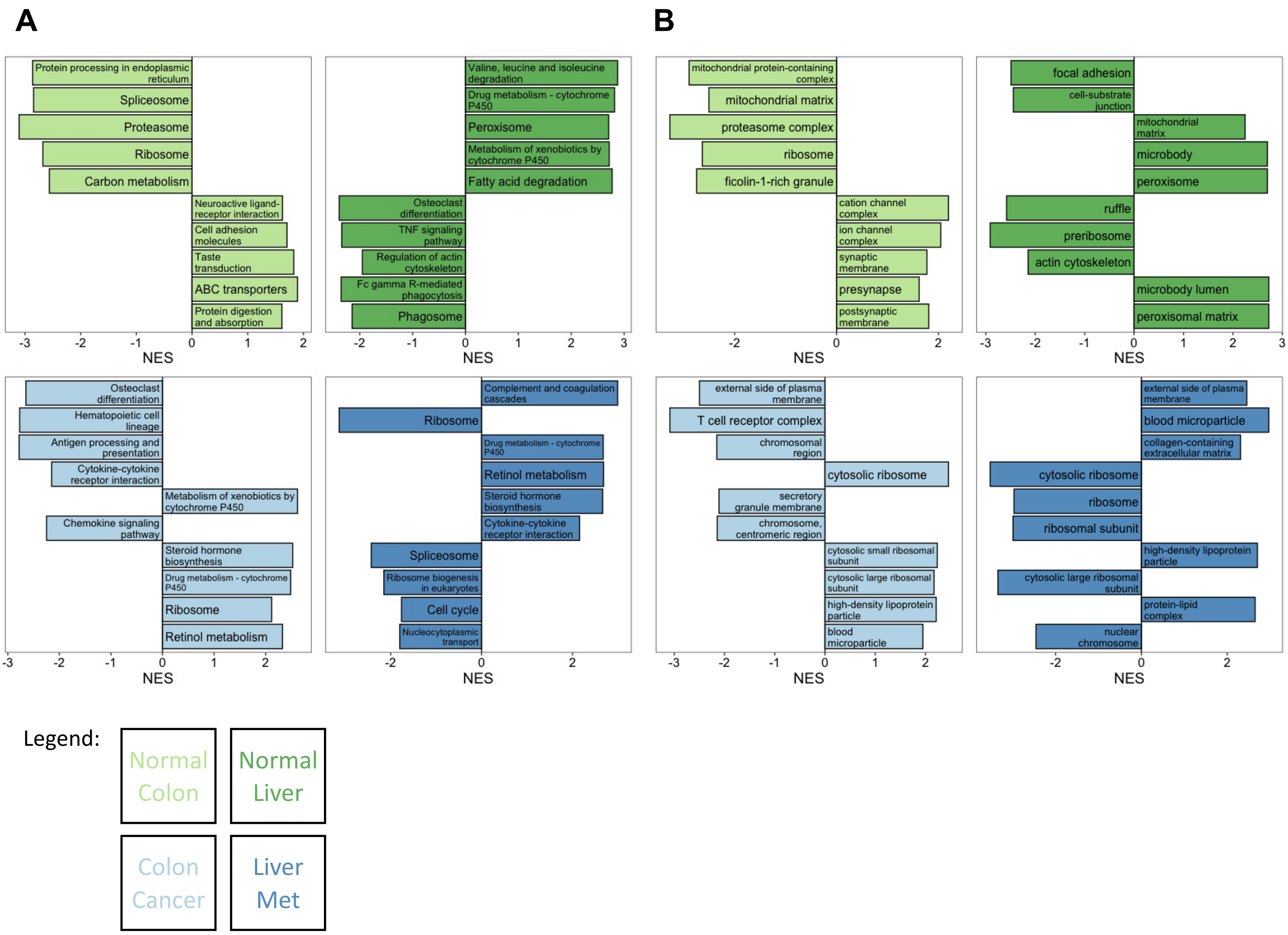

Figure S3 Gene set enrichment analysis of genes correlated with *SMLR1* mRNA expression

A Gene set enrichment analysis results based on the KEGG database among tissues.

B Gene set enrichment analysis results based on cellular component gene ontology database among tissues.

Top-left (light green): Normal colon; Top-right (dark green): Normal liver; Bottom-left (light blue): Primary colon cancer; Bottom-right (dark blue): Liver metastasis of colon cancer.

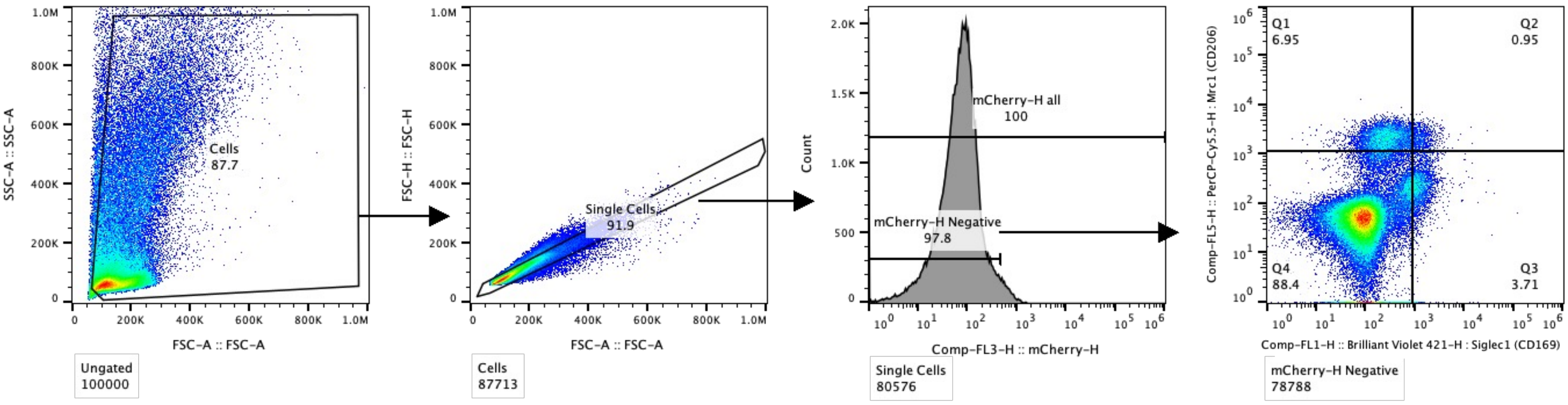

Figure S4 Gating strategy of liver resident cells

The gating strategy that had been applied to all samples. Cancer cells were engineered and labeled with mCherry.

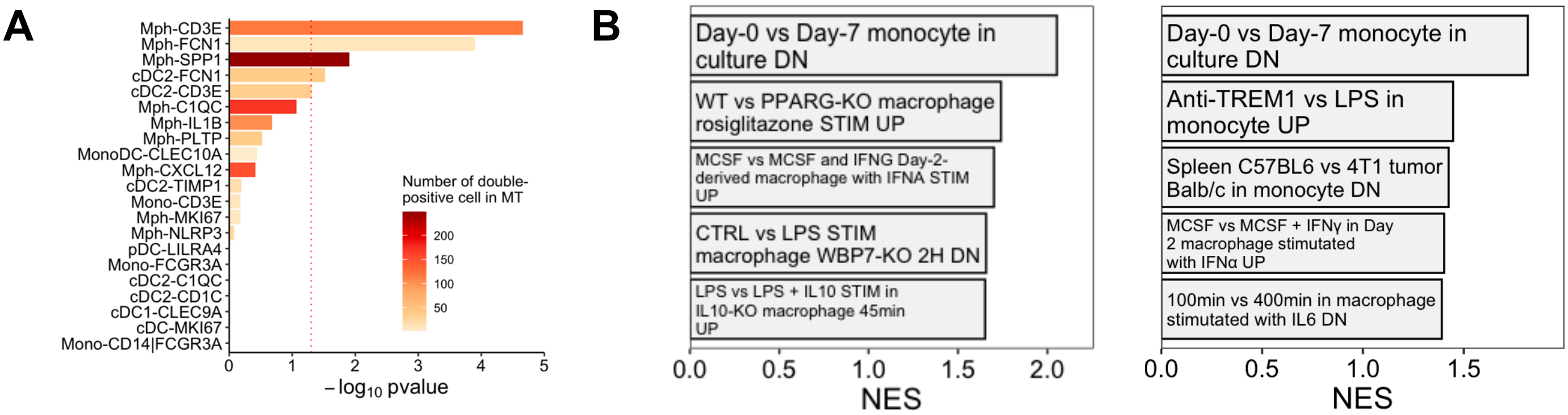

Figure S5 Characterization of *SIGLEC1⁺MRC1⁺* cells in CRLM tissues

A Comparison of the proportion of *SIGLEC1*$^{+}$*MRC1*$^{+}$ cells between liver metastasis of colon cancer and adjacent normal liver by Fisher exact test.

B Gene set enrichment analysis (GSEA) of immune signatures that enriched in *SIGLEC1*$^{+}$*MRC1*$^{+}$ cells compared with *SIGLEC1*$^{-}$*MRC1*$^{-}$ cells (left) and GSEA of immune signatures that enriched in *SIGLEC1*$^{+}$*MRC1*$^{+}$ cells compared with cells that are either *SIGLEC1*$^{+}$ *or MRC1*$^{+}$ (right).

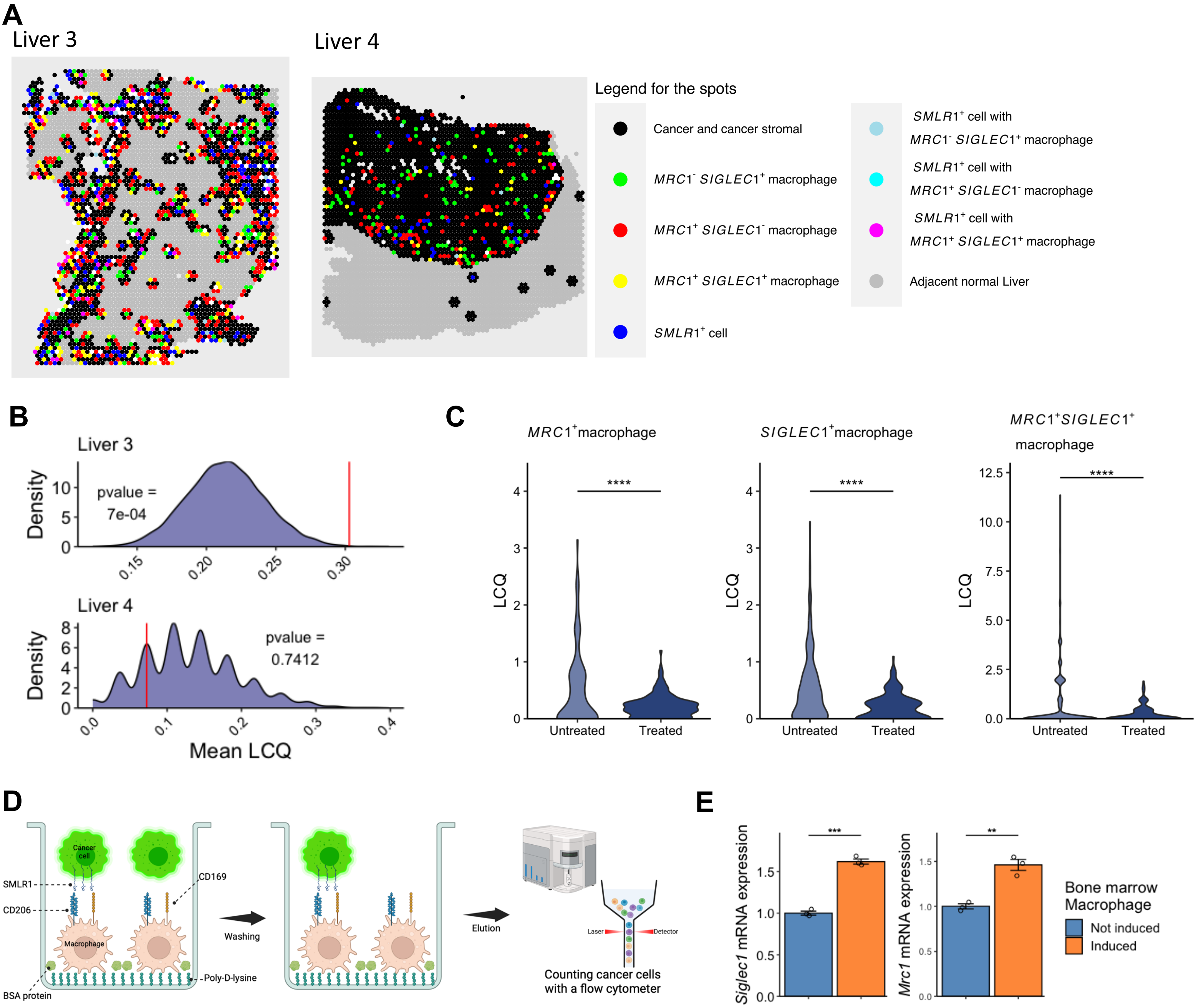

Figure S6 Cancer cell-macrophage interaction among patients and *in vitro* models

A Spatial transcriptomics of colon cancer liver metastasis tissues from patients treated with XELOX before the tumor resection. Left section: Liver 3; Right section: Liver 4.

B Quantification of local colocation quotient (LCQ) between spots that contain *SMLR1*-expressing cancer cells and *SIGLEC1-MRC1* double positive macrophage. Simulated null mean LCQ were plotted as density plot and mean LCQ for Liver 3 (top) and Liver 4 (bottom) were indicated by the red vertical line.

C Quantification of *SMLR1*-expressing cancer cell and indicated cell types colocalization in colon cancer liver metastasis among neoadjuvant treated and untreated patients.

D Schematic diagram illustrating the steps of the cell retention assay.

E *Siglec1* and *Mrc1* gene expression among mouse bone marrow macrophages upon IL4 induction (n = 3).

Data information: Data are presented as mean ± SEM. *p-value < 0.05, **p-value < 0.01, ***p-value < 0.001, and ****p-value < 0.0001.
